# Accurate alpha-particle stopping power measurements in graphenic carbon foils and their application to high-precision, non-destructive areal density determination

**DOI:** 10.1038/s41598-026-57488-0

**Published:** 2026-06-14

**Authors:** Konstantina Botsiou, Sivaji Purushothaman, Hans Geissel, Timo Dickel, Joachim Enders, Emma Haettner, David J. Morrissey, Maxim Saifulin, Christoph Scheidenberger, Marilena Tomut, Helmut Weick, Jianwei Zhao

**Affiliations:** 1 GSI Helmholtzzentrum für Schwerionenforschung, Planckstraße 1, 64291 Darmstadt, Germany; 2 II. Physikalisches Institut, Justus-Liebig Universität Gießen, Heinrich-Buff-Ring 16, 35392 Gießen, Germany; 3 Department of Physics, Institute for Nuclear Physics, Technische Universität Darmstadt, Schlossgartenstr. 9, 64289 Darmstadt, Germany; 4 Helmholtz Forschungsakademie Hessen für FAIR (HFHF), Campus Darmstadt, Schlossgartenstr. 2, 64289 Darmstadt, Germany; 5 Department of Chemistry, Michigan State University, 48824 East Lansing, MI USA; 6 Helmholtz Forschungsakademie Hessen für FAIR (HFHF), Campus Gießen, Heinrich-Buff-Ring 16, 35392 Gießen, Germany; 7 Institute of Materials Physics, Universität Münster, Wilhelm-Klemm-Str. 10, 48149 Münster, Germany; 8 School of Physics, Beihang University, 100191 Beijing, China

**Keywords:** Chemistry, Materials science, Nanoscience and technology, Physics

## Abstract

A precise, non-destructive method for determining the areal density of thin graphenic carbon (GC) foils via alpha-particle energy loss is presented. Two types of GC foils — sourced from KETEK GmbH and Applied Nanotech Inc. — were investigated using a three-isotope mixed alpha source emitting particles in the 5.0–5.8 $\textrm{MeV}$ range. Both foils have similar nominal areal densities of approximately $0.2\,\mathrm {mg\,cm^{-2}}$, but differ slightly in chemical composition and microstructure. High-resolution alpha spectroscopy yielded energy-loss measurements with relative uncertainties below 1%. The uncertainty of the extracted areal densities and stopping powers is dominated by the determination of foil mass, area and composition metrology, rather than by the alpha-energy-loss measurement itself. Experimental stopping powers were obtained by combining the measured energy loss with independently determined foil masses and areas, and were compared with established stopping-power models. A modified Bethe formalism incorporating Barkas and Bloch corrections, together with an empirically adjusted mean excitation energy $I_\textrm{adj}$, provided the most consistent description of the data across the investigated energy range. The resulting values were $(73 \pm 2)\,\textrm{eV}$ for the KETEK foil and $(85 \pm 3)\,\textrm{eV}$ for the Applied Nanotech foil. The fitted stopping-power curves indicate a systematic difference between the two GC foils, consistent with their differing compositions and microstructures. Because the stopping-power model is calibrated against the same reference foils, however, this interpretation is model-dependent and requires further validation using independently characterised samples. While the method is well suited to thin foils, angular straggling and the non-linear energy dependence of the stopping power may limit its applicability beyond the thin-target approximation. The reported stopping-power data are relevant for benchmarking Monte Carlo simulations and modelling energy deposition in carbon-based materials, with applications in accelerator technology and radiopharmaceutical research. In medical physics, stopping power is closely related to linear energy transfer, which governs the biological effectiveness of alpha-emitting isotopes in targeted therapies.

## Introduction

The interaction of charged particles with matter has been a subject of interest since the early days of atomic and modern physics^1–6^. As a natural consequence, experiments involving ion-beam interactions with matter were found to be strongly influenced by the physical properties of the target material such as areal density, temperature, and structural state (e.g., gaseous, liquid, amorphous, crystalline). These properties determine the energy loss, energy loss straggling, and angular straggling experienced by an ion beam traversing the material. Vacuum windows^7^ and stripper foils^8–10^, both common components of accelerator systems, are typical examples where such effects are critical. In turn, the interaction of the beam with the atoms of the foils has a decisive influence on the material properties; their stability, and resistance to deformation are therefore essential considerations, especially at high fluence. Another example is the energy loss of primary or secondary beams in production targets and degraders at in-flight radioactive-ion-beam facilities.

With the rapid advancement of materials science and engineering, a new class of graphenic carbon (GC) foils has emerged, and initial tests of these materials indicate promising potential for various applications^11–14^. These materials are being explored as alternatives to conventional beam windows, particularly where minimal areal density is required. In such applications, low-atomic-number (low-$Z$) materials are preferred to reduce energy loss and multiple scattering of heavy-ion beams^7,15,16^. Carbon-based foils are also widely used as stripper foils in particle accelerators to increase the charge state of ions and thereby improve acceleration efficiency. In addition to accelerator applications, ultra-thin carbon stripper foils are used in time-of-flight mass spectrometers for space plasma instrumentation, where they enable charge conversion and timing of incoming particles^17^. Graphene and graphenic carbon foils have also been investigated for such low-mass foil applications because of their favorable transmission, scattering, and thermo-mechanical properties^18^. Their low atomic number, propetries, non-toxicity, and high stripping efficiency make them especially suitable for operation under intense beam conditions. Among these, diamond-like carbon (DLC) foils represent the most established and extensively studied class, demonstrating excellent performance and operational lifetime^19,20^.

In this context, initial studies indicate that graphenic carbon (GC) foils may offer additional potential as an alternative or complementary stripper material, motivating their investigation in accelerator applications. This study focuses on commercially available GC foils that were jointly developed by KETEK GmbH and the Technical University of Munich using a novel production technique^11–14^. To enable methodological comparison and reproducibility, GC foils of similar areal density, produced using a different fabrication method, were also provided by Applied Nanotech^21^.

Proton energy-loss and transmission measurements are commonly used for determining the areal density of thin foils, particularly in accelerator-based studies where the incident beam energy, collimation, and geometry can be well controlled. Such methods provide a direct and well-established route to relate the measured energy loss to the amount of material traversed by the charged particles. However, they require access to a suitable accelerator beam and typically demand stringent control of beam energy stability, reproducibility, and experimental geometry.

This work investigates the stopping power and the accuracy of areal-density determination for GC foils produced by KETEK and Applied Nanotech^22–24^, using alpha-particle spectroscopic techniques. The use of alpha-particle energy loss provides an alternative, compact, and well-established approach for determining the areal density of thin foils^25–30^. A mixed alpha source offers several discrete and stable particle energies within a single laboratory setup, reducing the experimental complexity compared with accelerator-based proton measurements.

The accuracy of both proton- and alpha-particle energy-loss methods depends on the stopping-power model employed in the analysis. Since the measured energy loss is converted into areal density using theoretical or semi-empirical stopping-power values, uncertainties or limitations in these models can directly affect measurement precision, particularly for low-Z, nanostructured, or novel materials such as graphenic carbon (GC).

In contrast to protons, the use of alpha particles requires consideration of possible charge-exchange effects. In the $\textrm{MeV}$ energy range relevant to this work and in the thin-foil Vavilov regime^31^, alpha particles traverse solid carbon with an effective charge very close to $Z = 2$. Under these conditions, charge-exchange effects are not expected to contribute measurably to the observed energy loss. In this study, stopping power is evaluated using a modified Bethe formalism, which incorporates higher-order corrections and an empirically adjusted mean excitation energy $(I_{\textrm{adj}})$, enabling precise modeling of the experimental data. Any residual charge-state effects are accounted for through the inclusion of Barkas and Bloch corrections in the stopping-power model used in this work.

A three-isotope mixed alpha source that emits several well-identified alpha lines was used. The availability of multiple discrete energies within a single source makes the energy-loss calibration more reliable and enables energy-dependent evaluation of stopping power. Furthermore, careful spectral analysis, including the treatment of peak asymmetries, contributes significantly to the precision and reliability of the measurements. Beyond its scientific relevance, accurate knowledge of alpha-particle stopping power in absorber materials is essential for applications in both industry and medicine.

The 5.0–5.8 $\textrm{MeV}$ alpha-particle energy range is well suited for GC foils with areal densities around $0.2\,\mathrm {mg\,cm^{-2}}$, because it produces measurable energy losses of approximately 140–153 $\textrm{keV}$ while preserving well-resolved transmitted alpha peaks for precision centroid determination.

## Methods

### Energy-loss measurements of alpha particles

High-resolution alpha-particle energy measurements were performed using a three-isotope mixed, unsealed alpha calibration source containing $^{239}$Pu, $^{241}$Am, and $^{244}$Cm (Eckert & Ziegler, Composite Alpha Source; see Ref^32^.), which emit well-defined alpha lines in the energy range from 5.0 to 5.8 $\textrm{MeV}$ (see Table 2).

To verify the energy calibration, an unsealed $^{225}$Ac recoil-ion source prepared in the laboratory was measured using the same detector calibration established with the aforementioned three-isotope mixed source. The alpha-particle energies of $^{225}$Ac and its short-lived daughters ($^{221}$Fr, $^{217}$At, and $^{213}$Po) agreed with literature values to within 2 $\textrm{keV}$, well within the quoted uncertainties. No systematic energy offsets or additional energy losses were observed. This independent cross-check confirms that neither source-related self-absorption nor unaccounted window effects significantly impact the measurements, thereby validating the reliability of the $\Delta E$-based stopping power and areal-density determinations.

A dedicated vacuum-chamber setup was employed to measure the energies of alpha particles before and after transmission through graphenic carbon (GC) foils using a silicon surface-barrier detector (ORTEC Ultra BU-014–150-500). The alpha source was mounted on the top flange of the chamber with a source-to-detector distance of 55 mm, while the GC foil was positioned 38 $\textrm{mm}$ below the source. The alpha-particle beam was defined using a circular collimator with a diameter of 2 mm, positioned 29.7 $\textrm{mm}$ downstream of the 7 $\textrm{mm}$ diameter source and 8.3 $\textrm{mm}$ upstream of the foil. Based on this geometry, the illuminated footprint on the foil has a geometric outer diameter of approximately 4.5 $\textrm{mm}$.

All measurements were performed under high-vacuum conditions of approximately $10^{-6}$ $\textrm{mbar}$, maintained using a turbomolecular pumping system. Under these conditions, energy loss and straggling due to interactions with residual gas are negligible compared to the experimental uncertainties. Each reported areal density therefore represents a spatial average over the illuminated region of the foil. As a result, any surface roughness or local thickness variations are intrinsically averaged over the beam spot and do not bias the mean energy-loss value. Possible non-uniformities within the illuminated area would primarily manifest as additional peak broadening rather than as a systematic shift of the peak centroid.

Detector signals were processed through a charge-sensitive preamplifier (ORTEC 142 B), shaping amplifier (ORTEC 572), and multichannel analyzer (ORTEC MCB 926). Pulse-height spectra were recorded using ORTEC Maestro software. Linearity of the system was tested using a pulse generator, confirming 0.03% linearity in the relevant energy range and a high signal-to-noise ratio ($\sim$200:1).

The observed spectrum corresponds to the convolution of the true energy-loss distribution with the detector response. The detector energy resolution was determined to be $\textrm{FWHM} = 16.5\,\textrm{keV}$, corresponding to a Gaussian width of $\sigma _{\textrm{det}} \approx 7.0\,\textrm{keV}$. Convolution with a symmetric Gaussian does not shift the centroid; therefore, the detector resolution does not bias the measured $\Delta E$, although it increases the statistical uncertainty. This broadening was explicitly included in the fit model used to extract $\Delta E$.

The stopping power of a material describes the energy lost by a charged particle as it traverses a medium. It is commonly separated into electronic stopping, arising from excitation and ionization of target electrons, and nuclear stopping, arising from elastic interactions with target nuclei. In the $\textrm{MeV}$ alpha-particle energy range investigated here, the energy loss in thin carbon foils is dominated by electronic stopping; nuclear stopping is much smaller and is included only where provided by the tabulated or semi-empirical stopping-power models used for comparison. When the relative energy loss $\Delta E/E_i$ exceeds 3%, the nonlinear energy dependence of the stopping power within the foil must be considered, and the measured loss is typically assigned to an effective energy rather than to a simple arithmetic mean^33^. In the present case, the maximum relative energy loss was less than 3%; therefore, such nonlinear corrections are considered unnecessary^34–36^. The experimental stopping-power values *S* reported here were determined directly from the measured absolute energy loss and the independently measured areal density as,

1 $$\begin{aligned} S = \frac{\Delta E}{\rho _a}, \end{aligned}$$

where $\Delta E = E_i - E_f$ is the absolute energy loss of the alpha particle, with $E_i$ and $E_f$ denoting the initial and final energies of the alpha particle before and after passing through the foil, respectively, and $\rho _a$ is the measured areal density in units of mg cm$^{-2}$. *S* is expressed in units of $\mathrm {keV\,cm^{2}\,mg^{-1}}$.

The stopping power is evaluated at the arithmetic mean of the initial and final projectile energies,

2 $$\begin{aligned} E_m = \frac{E_i + E_f}{2} = E_i - \frac{\Delta E}{2}, \end{aligned}$$

which represents the average particle energy within the foil.

With the measured areal densities and alpha energies, the energy-loss straggling can be characterized by the Vavilov parameter, $\kappa$^31^,

3 $$\begin{aligned} \kappa = \frac{\xi }{E_{\max }}, \qquad \xi = \frac{K}{2}\,Z_1^{2}\,\frac{Z_2}{A_2}\,\frac{\rho _a}{\beta ^{2}}, \qquad E_{\max } \approx 2 m_e c^2 \beta ^{2}\gamma ^{2}, \end{aligned}$$

where $K = 0.307075~\mathrm {keV\,cm^{2}\,mg^{-1}}$, $\rho _a$ is the areal density, $Z_1$ is the projectile charge, $Z_2$ and $A_2$ are the atomic number and mass of the target material, and $\beta ,\gamma$ are obtained from the alpha-particle energy.

Using this expression, $\kappa$ decreases with energy from $\kappa \approx 7.6$ to 5.9 for the KETEK foil ($\rho _a = 0.185$ mg cm$^{-2}$) and from $\kappa \approx 7.9$ to 6.1 for the Applied Nanotech foil ($\rho _a = 0.193$ mg cm$^{-2}$). These values place the data firmly in the *Vavilov regime*, intermediate between Landau and Gaussian. In this regime, the intrinsic distribution is only mildly asymmetric, and the centroid obtained from convolution with the detector response provides a robust measure of $E_f$ for stopping-power determination.

### Validation via mass and area measurements

Each GC foil from KETEK is supported by a silicon ring that allows easy integration into potential applications but weighs approximately 2000 times more than the freestanding foil itself. Thus, direct weighing of the samples would introduce significant uncertainties.

To validate the non-destructive energy-loss-based areal-density method, two foils–one from KETEK and one from Applied Nanotech–were selected for destructive analysis using conventional mass and area determination. Sample masses were determined using a METTLER TOLEDO UMX2 ultra-microbalance. For each foil, nine repeated measurements were performed and averaged to obtain the mean values reported in Table 1. The standard deviation of these repeated measurements was $0.0001\,\textrm{mg}$, characterizing the repeatability of the weighing procedure.

The uncertainties reported in Table 1 represent the combined mass uncertainty, which includes both the statistical repeatability and the intrinsic calibration uncertainty of the balance. According to the manufacturer’s calibration certificate, the absolute uncertainty (one standard deviation) in the sub-milligram range is approximately $0.00018\,\textrm{mg}$. For the foil masses investigated in this work, this calibration uncertainty constitutes the dominant contribution to the total mass uncertainty.

Foil areas were determined using high-resolution optical microscopy (Keyence VHX6000) and edge detection implemented in the ImageJ software^37^. The areas were extracted from microscope images using calibrated pixel-to-length scaling factors provided by the microscope software. Different microscope magnifications were employed during image acquisition. Areal values were obtained by converting the measured pixel areas using the square of the respective linear scaling factor. Each area measurement was repeated 20 times to obtain reliable mean values and statistical uncertainties. The relative uncertainty of the pixel-to-length scaling factors was reported to be below 1%, nevertheless, to ensure a conservative uncertainty estimate, a relative uncertainty of 1% was included in the determination of the foil area. No measurable optical nonlinearities across the image field were observed, and their contribution to the area uncertainty was considered negligible.

The mass and area results are summarized in Table 1. From the measured mass and area, the areal density was calculated and used as a reference for benchmarking. The areal densities determined from mass and area (see Table 1) were subsequently used to derive stopping powers from the observed energy losses, enabling benchmarking of stopping-power models and extraction of the adjusted mean excitation energy.

**Table 1:** Areal density values obtained from direct mass and area measurements and from the alpha-particle energy-loss method for two graphenic carbon (GC) foils. The close agreement demonstrates internal consistency of the approach for the two reference foils investigated here, but does not constitute full external validation of the method across different graphenic carbon materials.

**Foil Manufacturer**	**Mass [** $\textrm{mg}$ **]**	**Area [** $\mathrm {cm^{2}}$ **]**	**Areal Density [** $\mathrm {mg\,cm^{-2}}$ **]**	
			**(mass/area)**	**(energy loss)**
KETEK	$0.0521 \pm 0.0002$	$0.281 \pm 0.006$	$0.186 \pm 0.004$	$0.185 \pm 0.001$
Applied Nanotech	$0.0382 \pm 0.0002$	$0.198 \pm 0.004$	$0.193 \pm 0.004$	$0.193 \pm 0.001$

### Data analysis

**Fig. 1:**
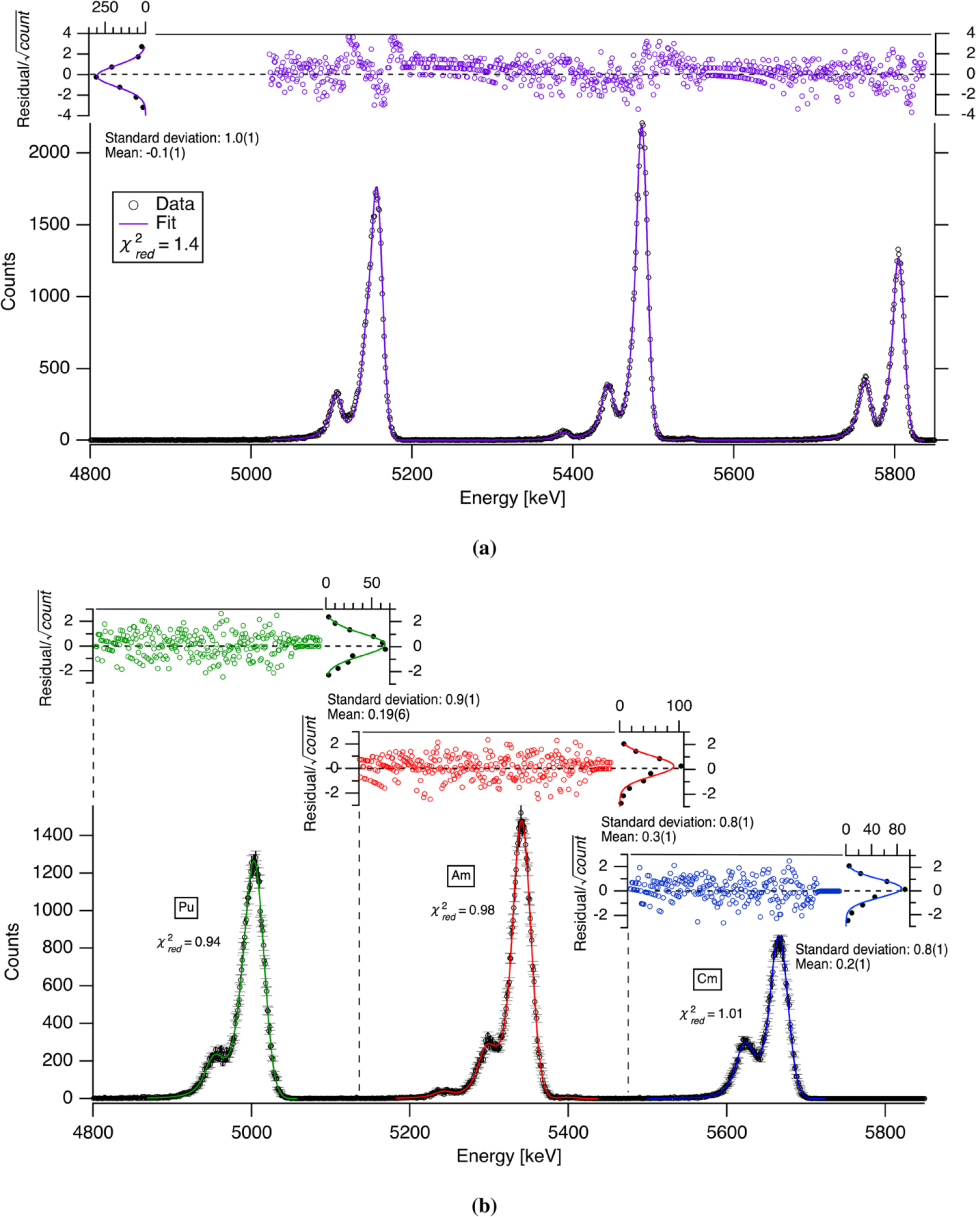
Energy-calibrated alpha spectra from a $^{239}$Pu, $^{241}$Am, and $^{244}$Cm mixed source: **(a)** bare spectrum; **(b)** energy-loss spectrum after transmission through the GC foil. The spectra were fitted using a Gaussian–exponential mixture model by least-squares minimization. The reduced chi-squared values, $\chi ^2_{\textrm{red}}$, are indicated for each fit. The normalized residuals show minor structures in low-statistics regions due to finite bin width effects.

Figure 1 presents a representative example of the analysis of the measured alpha-particle spectra and their residuals. Panel (a) shows the bare three-isotope source spectrum, measured without the GC foil between the source and the detector, while panel (b) shows the corresponding spectrum after transmission through the GC foil.

The recorded spectra were converted from ADC channels to energy using a linear calibration based on literature alpha-particle energies. The calibrated spectra were then analyzed by peak-shape fitting to account for the characteristic low-energy tailing arising from incomplete charge collection and scattering processes.

Each alpha-peak was modeled using a two-component mixture (i.e., a weighted sum) of Gaussian–exponential (GE) functions, originally proposed by Routti et al.^38^, to describe both the central peak and the low-energy exponential tail. The two components share a common Gaussian width but differ in their exponential decay parameters.

The fit function includes all alpha lines of $^{239}$Pu, $^{241}$Am, and $^{244}$Cm with branching ratios $\ge 0.2\%$. For the stopping-power and areal-density analysis, the dataset was restricted to lines with branching ratios $>1\%$, which are listed in Table 2. An exception is the 5144.3 $\textrm{keV}$ line of $^{239}$Pu (17.11%), which lies within the experimental resolution of the stronger 5156.59 $\textrm{keV}$ line (70.77%). This weaker line was included in the fit model but treated as unresolved and therefore excluded from the subsequent stopping-power analysis.

**Table 2:** Comparison of alpha-particle stopping power for KETEK and Applied Nanotech GC foils using measured areal densities. Isotope and branching ratio (BR) information for the alpha lines are included. The sources of alpha-particle energies are the National Nuclear Data Center (NNDC) database^39^ and the Live Nuclear Chart from the International Atomic Energy Agency (IAEA)^40^. Mean energy $E_m$ and stopping power $S_{\text {exp}}$ were derived from measured energy loss $\Delta E$ values.

**Isotope**	**BR (%)**	$E_i$ [$\textrm{keV}$]	**KETEK**, $\rho _a = 0.186 \pm 0.004\,\mathrm {mg\,cm^{-2}}$			**Nanotech**, $\rho _a = 0.193 \pm 0.004\,\mathrm {mg\,cm^{-2}}$		
			$-\Delta E$ [$\textrm{keV}$]	$E_m$ [$\textrm{keV}$]	$S_{\text {exp}}$ [$\mathrm {keV\,cm^{2}\,mg^{-1}}$]	$-\Delta E$ [$\textrm{keV}$]	$E_m$ [$\textrm{keV}$]	$S_{\text {exp}}$ [$\mathrm {keV\,cm^{2}\,mg^{-1}}$]
^239^Pu	11.94	$5105.5 \pm 0.8$	$152.9 \pm 0.7$	$5029 \pm 1$	$822 \pm 19$	$152.5 \pm 1$	$5029 \pm 0.9$	$790 \pm 17$
^239^Pu	70.77	$5156.6 \pm 0.1$	$151.8 \pm 1.0$	$5081 \pm 1$	$816 \pm 19$	$151.4 \pm 1$	$5081 \pm 1.1$	$785 \pm 17$
^241^Am	1.66	$5388.0 \pm 0.1$	$146.2 \pm 1.0$	$5315 \pm 1$	$786 \pm 18$	$146.6 \pm 1$	$5315 \pm 1.3$	$760 \pm 17$
^241^Am	13.1	$5442.8 \pm 0.1$	$146.2 \pm 0.7$	$5370 \pm 1$	$786 \pm 18$	$145.8 \pm 1$	$5370 \pm 0.9$	$755 \pm 16$
^241^Am	84.8	$5485.6 \pm 0.1$	$144.6 \pm 0.6$	$5413 \pm 1$	$777 \pm 18$	$145.0 \pm 1$	$5413 \pm 0.8$	$751 \pm 16$
^244^Cm	23.1	$5762.64 \pm 0.03$	$140.2 \pm 0.6$	$5693 \pm 1$	$754 \pm 17$	$139.6 \pm 1$	$5693 \pm 0.9$	$723 \pm 16$
^244^Cm	76.9	$5804.8 \pm 0.1$	$139.0 \pm 0.6$	$5735 \pm 1$	$747 \pm 17$	$140.0 \pm 1$	$5735 \pm 0.8$	$725 \pm 16$

Least-squares fitting was performed in Igor Pro 8^41^, yielding peak centroids, full widths at half maximum (FWHM), and statistical uncertainties. The goodness of fit was evaluated using the chi-squared ($\chi ^2$) statistic to assess the consistency between the model and the measured spectra. The fit to the bare spectrum in Figure 1(a) yields normalized residuals that follow an approximately normal distribution, with small systematic deviations confined to low-count regions between peaks and at the leading edge of the $^{239}$Pu peak. Figure 1(b) shows the spectrum after transmission through the GC foil. The $^{239}$Pu (green), $^{241}$Am (red), and $^{244}$Cm (blue) peaks were fitted simultaneously using common shape parameters, while the energy loss $\Delta E$ of each alpha line was treated as a free parameter. The value of $\Delta E$ was determined from the shift in centroid positions between spectra recorded with and without the GC foil in the beam path. The experimental stopping power was then calculated as the ratio of the measured energy loss to the independently determined foil areal density obtained from mass and area measurements (see Table 1).

Two primary sources of uncertainty were considered in the stopping-power determination: the uncertainty associated with energy-loss measurements and that related to the areal density of the foils. The energy-loss uncertainty included contributions from calibration errors, statistical uncertainties arising from peak fitting, and uncertainties in the literature reference values of the alpha energies. The overall uncertainty in the stopping power was calculated through standard error propagation, taking into account the relative contributions of energy-loss and areal-density uncertainties. The detector resolution was included through the peak-shape model and affects the statistical uncertainty of the centroid extraction, but does not shift the centroid for a symmetric response function.

For thin targets, such as the foils studied in this work, the interaction of charged particles with matter is conveniently described in terms of the *mass stopping power*, which directly relates the measured energy loss to the material properties of the absorber. For this purpose, the experimentally extracted stopping-power values were fitted using the Bethe formalism^42,43^,, including the Barkas^44^, and Bloch^45–47^, corrections. The mass stopping power *S* is defined as the mean energy loss per unit areal density and is given by

4 $$\begin{aligned} S = -\frac{1}{\rho _v}\frac{dE}{dx} = K \frac{Z_2}{A_2} \frac{Z_1^2}{\beta ^2} \left[ 1 - e^{-\frac{130\beta }{Z_1^{2/3}}} \right] ^2\left[ \left( 13.8373+ \ln \!\left( \frac{\beta ^2}{1-\beta ^2}\right) - \beta ^2- \ln (I_{\text {m}})-\frac{C}{Z_{2}^{2}}\right) L_{\textrm{Barkas}} + \Delta L_{\textrm{Bloch}} \right] , \end{aligned}$$

where $\rho _v$ is the mass density of the material, *dE* is the energy loss, and *dx* is the path length, such that $\rho _v\,dx = \rho _a$ corresponds to the areal density of the absorber. In this expression, $Z_1 = 2$ (He), $Z_2 = 6$, $A_2 = 12.0106$, and $K = 0.307075\,\mathrm {keV\,cm^{2}\,mg^{-1}}$. The terms $L_{\textrm{Barkas}}$ and $\Delta L_{\textrm{Bloch}}$ denote the Barkas and Bloch correction terms, respectively, while $C/Z_2^{2}$ represents the shell correction.

The mean excitation energy $I_{\text {m}}$ represents an average over the electronic excitation and ionization potentials of a material. Its precise value is difficult to determine due to limited knowledge of oscillator-strength distributions in the relevant energy range (10–1000) $\textrm{eV}$. In practice, $I_{\text {m}}$ is often derived from experimental stopping power data by inverting the Bethe formula to solve for $\ln (I_{\text {m}})$. Standard reference values for $I_{\text {m}}$ in carbon-based materials include $(78.0 \pm 7.0)$ eV for graphite and $(82 \pm 2)\,\textrm{eV}$for amorphous carbon, as recommended in ICRU Reports 37 and 49^48,49^, and a more recent value of $(77 \pm 4)\,\textrm{eV}$ for graphite based on optical and X-ray spectroscopy^50^.

However, the assumption that the shell correction term $C/Z_{2}^{2}$ in the Bethe model vanishes at high energies, i.e. $(C/Z_{2}^{2})_{\beta \rightarrow 1}=0$, is not strictly valid, particularly for light elements such as carbon. To account for such deviations, the NAS–NRC Subcommittee on Penetration of Charged Particles^51^ introduced the concept of an *adjusted mean excitation energy* $I_{\text {adj}}$, defined as

5 $$\begin{aligned} \ln (I_{\text {adj}}) = \ln (I_{\text {m}}) + \frac{C}{Z_{2}^{2}}. \end{aligned}$$

This formulation effectively absorbs unresolved shell and material-specific corrections into a single empirical parameter, enabling improved agreement between theoretical stopping-power expressions and experimental data.

In the present work, $I_{\text {adj}}$ was therefore treated as a fitting parameter within the modified Bethe framework including Barkas and Bloch corrections. This approach provides a physically consistent description of electronic stopping for $\textrm{MeV}$ alpha particles while allowing empirical adjustment of material-dependent effects. In this way, the analysis remains consistent with established stopping-power frameworks and enables direct comparison with previous studies.

By treating $I_{\textrm{adj}}$ as a free fit parameter, the data can be described using foil-specific effective stopping-power curves for the KETEK and Applied Nanotech samples. These fitted differences are discussed and interpreted in the context of the model framework below. The fit also enabled an inverse application: areal-density determination by alpha-particle energy loss. Once the stopping-power model had been parameterized and $I_{\textrm{adj}}$ extracted, the areal density was calculated from the measured energy losses for the additional GC foils. The areal density is calculated by integrating the inverse of the stopping-power function over the energy interval corresponding to the particle’s energy loss. This approach accounts for the energy dependence of the stopping power and improves accuracy. For practical implementation, the integral in this work was approximated numerically by dividing the energy range into small equal segments.

The experimental stopping-power data were analyzed to determine $I_{\text {adj}}$ using Eqs. (4) and (5). The KETEK foils are produced by chemical vapor deposition (CVD), specifically rapid thermal CVD using ultra-high-purity precursor gases, a fabrication route known to yield graphenic carbon with predominantly sp$^2$-hybridized bonding and very low levels of chemical impurities^14^. The analysis was therefore performed assuming pure carbon.

For the Applied NanoTech foil, a pure carbon composition cannot be assumed. Independent X-ray photoelectron spectroscopy (XPS) measurements by Pavlovsky and Fink^23^ reported an elemental composition of 89.6 at.% carbon, 8.8 at.% oxygen, and 1.6 at.% nitrogen for chemically reduced graphene oxide foils of the same production type. This non-stoichiometric composition was explicitly taken into account using Bragg additivity of the mass stopping power. In this weighted-sum approach, the foil is treated as a mixture of its constituent elements, and the total mass stopping power is given by

6 $$\begin{aligned} S_{\textrm{foil}} = \sum _i w_i S_i , \end{aligned}$$

where $w_i$ are the elemental mass fractions ($w_{\textrm{C}}=0.868$, $w_{\textrm{O}}=0.114$, $w_{\textrm{N}}=0.018$), and $S_i$ denote the corresponding elemental mass stopping powers. In this analysis, the adjusted mean excitation energies of oxygen ($I_{\text {adj}}(\text {O}) = 103\,\textrm{eV}$) and nitrogen ($I_{\text {adj}}(\text {N}) = 91\,\textrm{eV}$) were adopted from the compilation and discussion by Ahlen^6^, while the adjusted mean excitation energy of the carbon component was treated as a fit parameter. The Barkas ($L_{\textrm{Barkas}}$) and Bloch ($\Delta L_{\textrm{Bloch}}$) correction terms, as well as the stopping-power curves for oxygen and nitrogen using Equations (4) and (5), were first calculated numerically using *Mathematica 13.0* and subsequently fitted with parametric power-law functions for efficient implementation in the main fitting routine. These parametrizations reproduce the numerically computed correction terms with a relative accuracy better than $10^{-3}$. The effective adjusted mean excitation energy of the foil was then determined using Bragg additivity as,

7 $$\begin{aligned} \ln I_{\text {adj}}(\textrm{foil}) = \sum _i f_i \ln I_{\text {adj}}(i), \qquad f_i = \frac{w_i (Z_i/A_i)}{\sum _j w_j (Z_j/A_j)}, \end{aligned}$$

where $f_i$ are electron-fraction weights calculated from the atomic number $Z_i$ and mass number $A_i$ of the constituent elements, and $I_{\text {adj}}(i)$ denote the individual adjusted elemental excitation energies.

Before presenting the results, we note an important limitation of the interpretation. The measured final alpha-particle energies after transmission through the KETEK and Applied Nanotech foils are very similar over the investigated energy range of 5.1–5.8 $\textrm{MeV}$. Considered alone, this observation would naturally suggest that the effective stopping powers of the two foils are also similar. In the present analysis, however, the inferred stopping powers depend not only on the measured energy losses but also on the independently assigned areal densities, their uncertainties, and the assumed elemental composition, in particular the non-stoichiometric composition adopted for the Applied Nanotech foil from Pavlovsky and Fink^23^. Possible limitations in the destructive mass-and-area determination and in the assumed stoichiometry may therefore influence the extracted stopping-power difference. Within this model framework, the authors prefer the interpretation of material-dependent effective stopping powers, which leads to the results presented below. This interpretation should be understood as model dependent and not as an independently established material property. Independent measurements of composition and structure, for example by elastic backscattering spectrometry (EBS), elastic recoil detection analysis (ERDA), Rutherford backscattering spectrometry (RBS), Raman spectroscopy, atomic force microscopy (AFM), or transmission electron microscopy (TEM), would be required to confirm this interpretation.

## Results

**Fig. 2:**
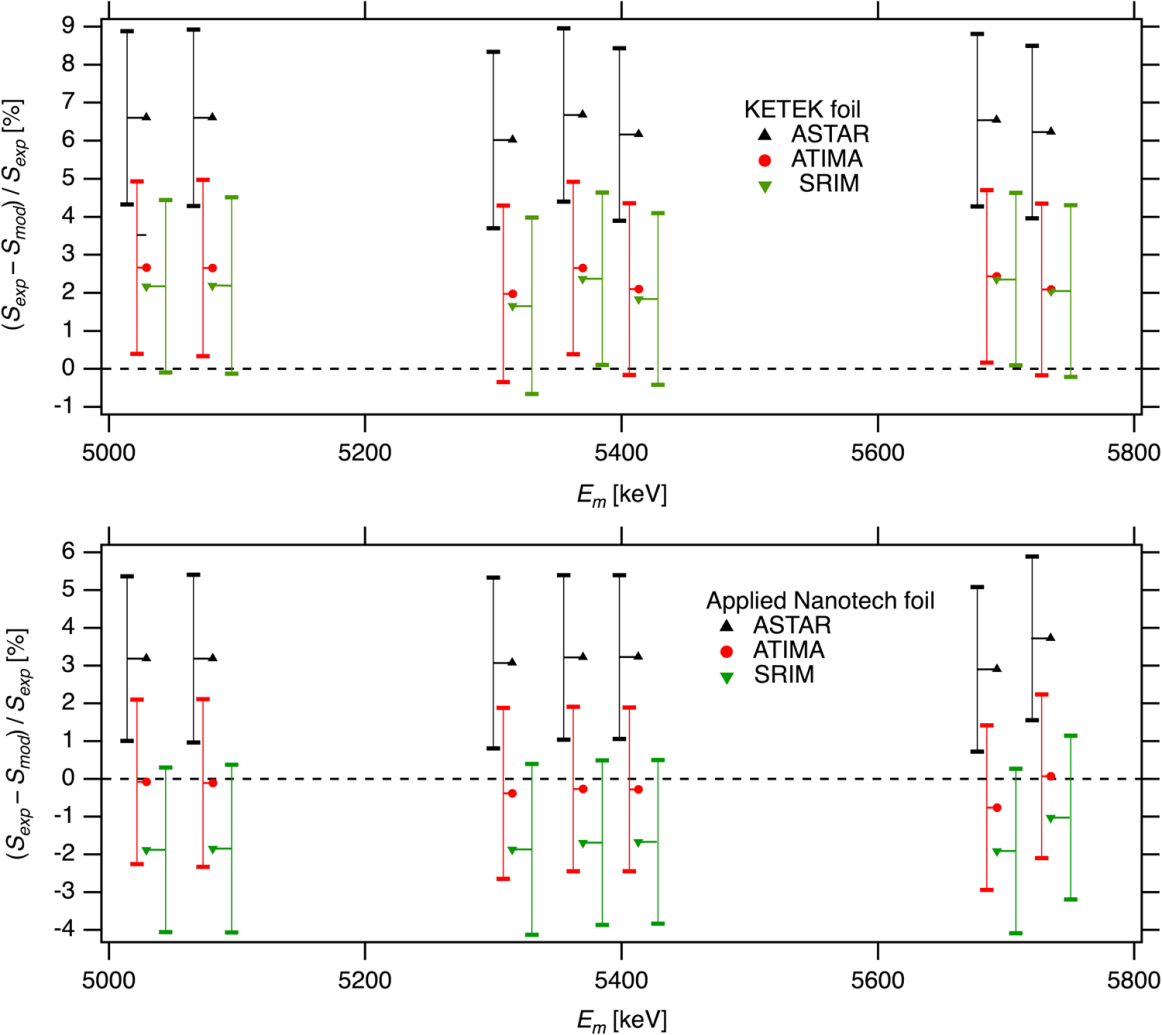
Relative deviation of stopping-power models (ASTAR, ATIMA 1.4^52^ and SRIM-2013^53,54^) from measured values for KETEK (top) and Applied Nanotech (bottom) GC foils as a function of mean energy $E_m$. For clarity, the data points were plotted with their error bars slightly offset horizontally to avoid overlap.

The experimentally determined stopping powers of the KETEK and Applied NanoTech GC foils were compared with widely used stopping-power models, including ATIMA 1.4^52^, SRIM-2013^53,54^, and ASTAR^55^, to assess the reliability of theoretical predictions in the investigated energy range. Figure 2 shows the relative deviation $(S_{\textrm{exp}} - S_{\textrm{mod}})/S_{\textrm{exp}}$ as a function of the mean $\alpha$-particle energy $E_m$, where $S_{\textrm{exp}}$ denotes the experimental stopping power and $S_{\textrm{mod}}$ the corresponding model value.

**Fig. 3:**
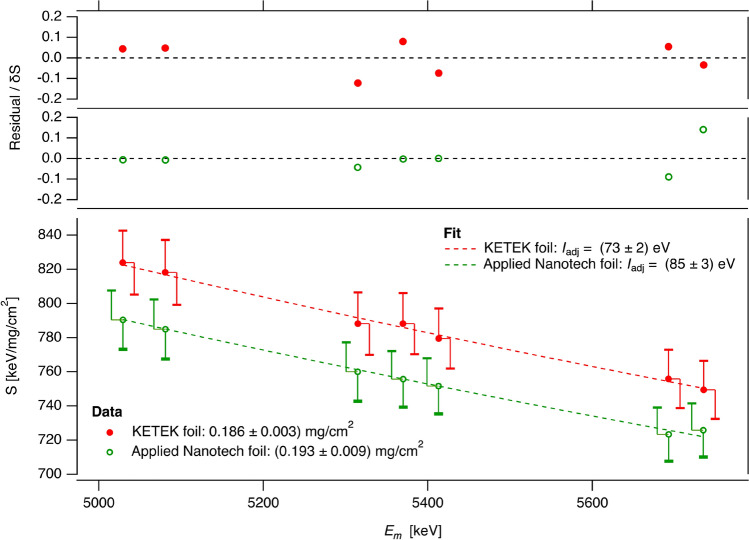
Experimental stopping power of alpha particles in graphenic carbon foils from KETEK (red filled circles) and Applied Nanotech (green open circles), shown as a function of the mean energy $E_m$. The data are fitted with the Bethe formula with Barkas and Bloch corrections, treating the mean excitation energy $I_\text {adj}$ as a free parameter. The dashed lines represent the corresponding best-fit curves, yielding material-specific $I_\text {adj}$ values (see Table 3). The middle and top panels display the normalized residuals (i.e., residuals divided by their respective uncertainties $\delta S$) for the KETEK and Applied Nanotech data, respectively. For clarity, the data points were plotted with their error bars slightly offset horizontally to avoid overlap.

**Fig. 4:**
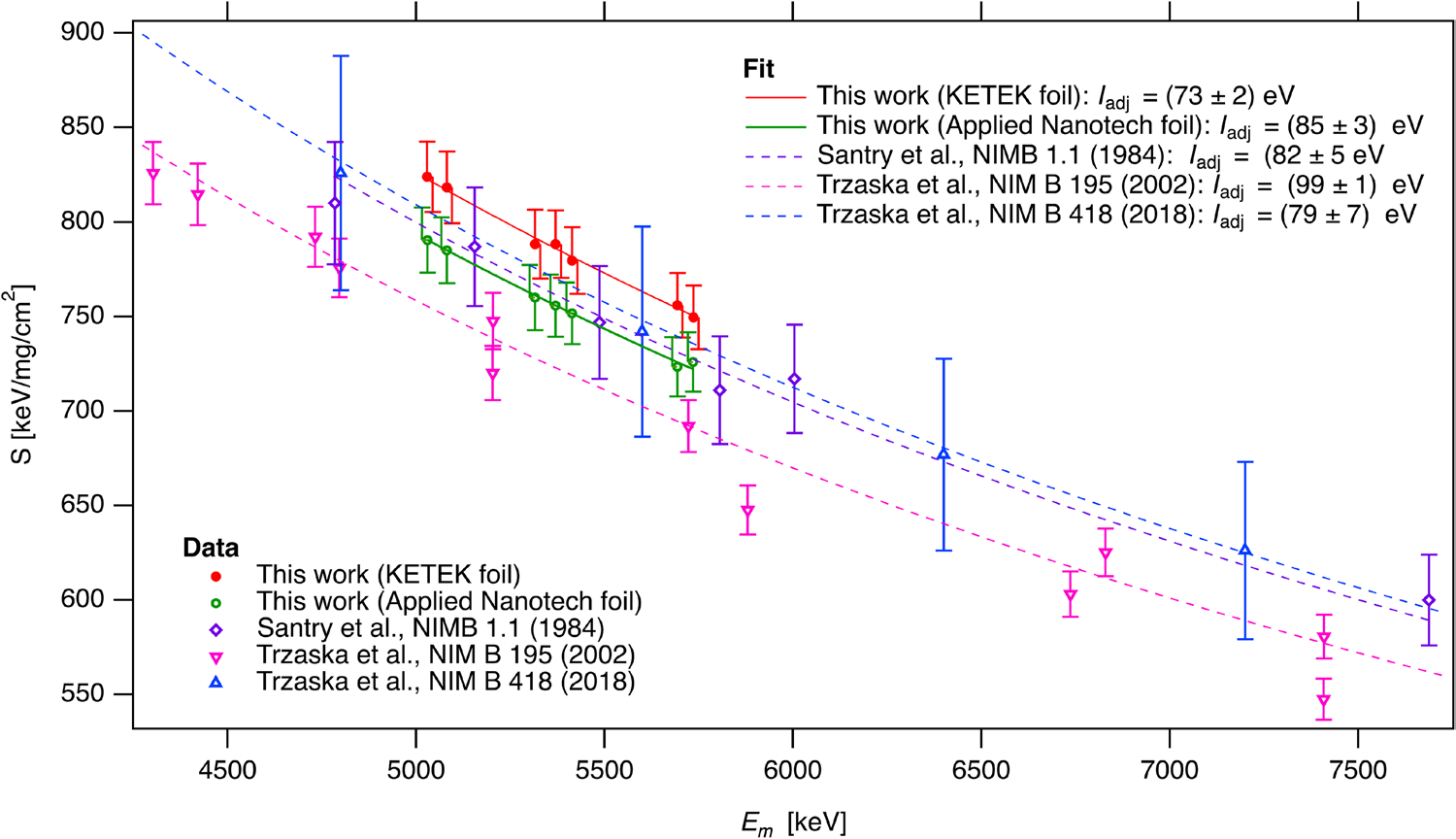
Mass stopping power of alpha particles in carbon as reported by Santry et al. (1984)^56^, Trzaska et al. (2002)^57^, and Trzaska et al. (2018)^58^, plotted as a function of the mean energy $E_m$. The dashed and dash-dotted curves represent fits using Eqs. (4) and (5), where the adjusted mean excitation energy $I_{\text {adj}}$ was treated as a free parameter for each dataset.

**Table 3:** Adjusted mean excitation energy $I_\text {adj}$ values extracted from experimental stopping-power data using the Bethe formalism with Barkas and Bloch corrections. Results for GC foils from KETEK and Applied Nanotech are compared with literature values. For the non-stoichiometric Applied Nanotech foil, the quoted value refers to the compound; the carbon-equivalent value is $I_{\text {adj}}(\textrm{C}) = (83 \pm 3)\,\textrm{eV}$.

**Data**	**Target type**	$I_\text {adj}$ [$\textrm{eV}$]
This work	GC (KETEK foil)	$73\pm 2$
This work	GC (Applied Nanotech foil)	$85 \pm 3$
Santry et al.^56^	Not specified	$82 \pm 5$
Trzaska et al.^57^	Not specified	$99 \pm 1$
Trzaska et al.^58^	Not specified	$79 \pm 7$

For the KETEK foil (top panel), all models exhibit small positive deviations, indicating a systematic underestimation of the stopping power. The largest deviations are observed for ASTAR, while SRIM-2013 shows the smallest offset. For the Applied NanoTech foil (bottom panel), the deviations are smaller in magnitude; ATIMA 1.4 and SRIM-2013 predictions lie closer to zero and remain within experimental uncertainties.

ASTAR consistently underestimates the stopping power for both samples. Nevertheless, all model predictions remain compatible with the experimental data within the combined uncertainties over the full investigated energy range.

In the case of the KETEK foil, the analysis was performed assuming pure carbon. In contrast, the Applied Nanotech foils exhibit a non-stoichiometric composition that deviates from pure carbon due to residual oxygen- and nitrogen-containing functional groups^23^. For implementation in ATIMA 1.4, SRIM-2013 and ASTAR, these atomic fractions were incorporated using Bragg’s additivity rule^59^.

Figure 3 presents the experimental stopping-power values for both GC foils and their corresponding fits obtained using the Bethe-Bloch formalism with the extracted $I_\text {adj}$ values using Equations (4) to (7). For the KETEK foil, the extracted value is $I_{\text {adj}} = (73\pm 2)\,\textrm{eV}$. For the Applied NanoTech foil, the fitting procedure yields an adjusted mean excitation energy for the carbon component of $I_{\text {adj}}(\text {C}) = (83 \pm 3)\,\textrm{eV}$, which was then combined with the fixed oxygen and nitrogen contributions using Bragg additivity to obtain the effective foil value of $(85 \pm 3)$ eV. These results provide the basis for the non-destructive areal-density measurement approach presented in the following section.

Within the adopted model framework, the difference between the extracted $I_{\textrm{adj}}$ values for the two foils suggests a systematic variation in their effective electronic stopping behavior, which may reflect differences in composition and material structure.

The middle and top panels of Figure 3 further illustrate the agreement between the experimental data and the theoretical model by displaying the normalized residuals, defined as the deviation between measured and fitted stopping power divided by the experimental uncertainty ($\delta S$). These plots highlight the overall agreement between experiment and theory across the full energy range.

The normalized residuals serve as a diagnostic of the internal consistency of the adopted stopping-power description. Residuals without a systematic energy-dependent trend support the consistency of the fitted model over the investigated energy range, whereas persistent structured residuals would indicate possible deficiencies in the assumed composition, areal density, or stopping-power formalism.

Figure 4 presents a comparison between the present stopping-power results and previous measurements for carbon reported by Santry et al. (1984)^56^, Trzaska et al. (2002)^57^, and Trzaska et al. (2018)^58^. Equations (4) and (5) were also applied to the literature data from Santry et al.^56^ and Trzaska et al.^57,58^, restricting the analysis to the energy range (2000–10000) $\textrm{keV}$ to ensure the validity of the theoretical formulation. These datasets span a similar $\alpha$-particle energy range and were analyzed consistently within the same fitting framework. The extracted $I_{\text {adj}}$ values from these earlier works range from $(79 \pm 7)$ eV to $(99 \pm 1)$ eV (see Table 3). The best-fit $I_{\text {adj}}$ values from both the present and historical datasets are summarized in Table 3.

### Areal density

The motivation for this work includes the development of a precise, low-cost, and non-destructive technique to determine the areal density $(\rho _a)$ of thin graphenic carbon (GC) foils. Such foils, particularly those manufactured by KETEK GmbH via chemical vapor deposition (CVD) followed by silicon wafer etching, are intended for use in heavy-ion beam experiments where precise control of material thickness and density is critical. The KETEK production process specifies a thickness uniformity of 1%. For such foils, areal density $\rho _a$ is the central quantity for ion transmission because it represents the amount of material traversed per unit area. It is therefore more directly related to charged-particle energy loss, energy-loss straggling, angular straggling, and transmission than geometrical thickness alone. Foils with similar nominal thickness may exhibit different effective stopping behavior if their density, porosity, roughness, composition, or microscopic structure differs.

The areal density was derived from alpha-particle energy loss using

8 $$\begin{aligned} \rho _{a} = -\int _{E_i}^{E_i - \Delta E} \frac{dE}{S(E)}, \end{aligned}$$

where $E_i$ is the initial energy, $\Delta E$ is the measured energy loss of the alpha particles, and $S(E)$ (Equations 4 and 5) is the stopping power. Due to the complexity of the analytical form of $S(E)$, the integral was evaluated numerically by dividing the energy range into sufficiently small steps.

**Fig. 5:**
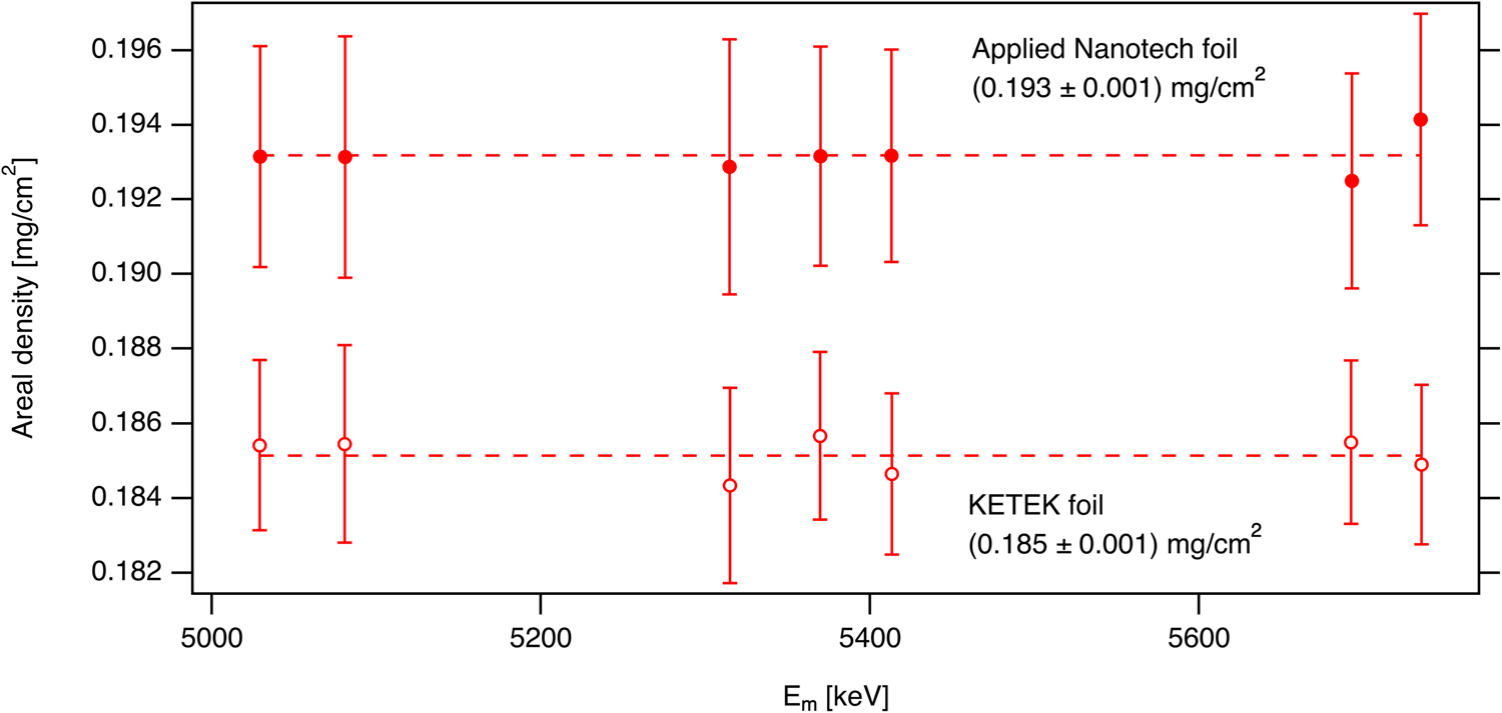
Areal density values for two graphenic carbon foils–Applied Nanotech (filled circles) and KETEK (open circles)–as a function of alpha-particle mean energy $E_m$. Each point corresponds to one resolved alpha line from the three-isotope mixed alpha source. The dashed lines represent the weighted mean of the measured data in each case.

Figure 5 shows the areal density values determined for two GC foils, Applied Nanotech and KETEK as a function of the alpha-particle mean energy $E_m$. Each data point corresponds to one of the resolved alpha lines used in the stopping-power and areal-density analysis. For each energy, the areal density was inferred by comparing the measured energy loss of alpha particles after transmission through the foil with the stopping power calculated using the modified Bethe formalism parameterized by the experimentally determined adjusted mean excitation energy $I_{\textrm{adj}}$.

The uncertainty of the areal-density values includes contributions from the statistical uncertainty of the energy-loss measurement and from the uncertainty of the stopping-power parameterization. The latter was evaluated by recalculating the areal density for $I_{\textrm{adj}} \pm \delta (I_{\textrm{adj}})$, with the corresponding half-difference taken as the standard uncertainty contribution associated with $I_{\textrm{adj}}$. Since $I_{\textrm{adj}}$ was extracted from the same dataset, this procedure accounts for the stopping-power model uncertainty without double counting contributions already reflected in the fitted parameter.

To obtain a reliable estimate of the foil areal density, values corresponding to the resolved alpha lines were combined using a weighted mean, such that measurements with smaller statistical uncertainty contribute more strongly. The resulting weighted mean areal densities are $(0.193 \pm 0.001)$ mg cm$^{-2}$ for the Applied Nanotech foil and $(0.185 \pm 0.001)$ mg cm$^{-2}$ for the KETEK foil, as indicated by the horizontal lines in Figure 5. The absence of a systematic energy dependence demonstrates the internal consistency of the non-destructive areal-density determination.

According to information provided by the manufacturer (private communication), the bulk density of the KETEK graphenic carbon (GC) foil was independently determined by measuring the thickness of representative films and performing X-ray transmittance measurements at the Physikalisch-Technische Bundesanstalt (PTB), Berlin. The measured transmittance data were compared with theoretical attenuation curves calculated using tabulated X-ray optical constants from the Center for X-ray Optics (CXRO) database^60^, yielding best agreement for a density in the range 2.1–$2.2\,\mathrm {g\,cm^{-3}}$. The nominal film thickness reported by the manufacturer is approximately $1\,\mu \textrm{m}$, with a quoted thickness uniformity of better than $1\%$ for an individual foil.

**Fig. 6:**
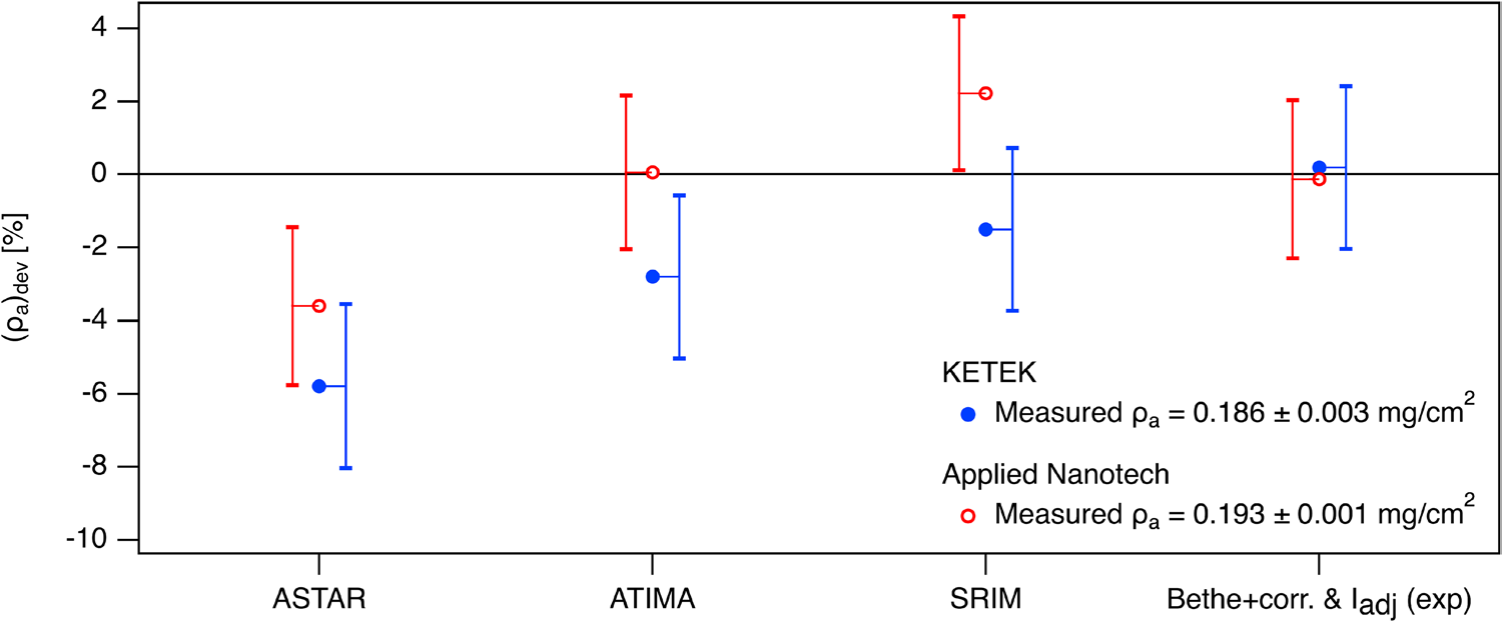
Relative deviation of areal-density estimates obtained using different stopping-power models with respect to the reference areal densities determined independently from mass and area measurements. Results are shown for GC foils from KETEK (filled symbols) and Applied Nanotech (open symbols). The best overall agreement is obtained using the modified Bethe formalism with Barkas and Bloch corrections and the experimentally determined adjusted mean excitation energies $I_{\textrm{adj}}$ from this work (see Table 3). For clarity, the data points were plotted with their error bars slightly offset horizontally to avoid overlap.

For the Applied Nanotech foils, manufacturer-provided information (private communication) indicates areal densities in the range of approximately 0.16–0.20 $\mathrm {mg\,cm^{-2}}$, with a quoted thickness uniformity of better than 5%. The experimentally determined areal densities obtained in this work fall within this range, demonstrating consistency between the non-destructive alpha-energy-loss measurements and independently available reference specifications.

To assess the model dependence of the areal-density determination, three stopping-power models were evaluated: SRIM-2013, ASTAR, and ATIMA 1.4. In each case, the areal density derived from the measured alpha-particle energy loss was compared with independently determined reference values obtained from mass and area measurements.

The relative deviations are summarized in Figure 6. The error bars represent the combined standard uncertainties of the areal-density determinations, including contributions from the area measurement, the mass determination, and the energy-loss method. For both KETEK (filled symbols) and Applied Nanotech foils (open symbols), SRIM-2013, ATIMA 1.4, and the modified Bethe formalism remain consistent with the reference value within the quoted uncertainties, whereas ASTAR exhibits a systematic underestimation that exceeds these uncertainties.

Overall, although the deviations are small, the residual spread among model predictions illustrates the sensitivity of precision areal-density determinations to the chosen stopping-power parameterization. Within uncertainties, the modified Bethe formalism including Barkas and Bloch corrections together with the experimentally determined $I_{\textrm{adj}}$ provides the most consistent agreement across both materials.

## Discussion

### Adjusted mean excitation energy and stopping-power differences

Accurate stopping-power measurements in carbon-based materials remain challenging due to the diversity of carbon allotropes and their distinct electronic structures. The stopping power of carbon is strongly influenced by its allotropic form; for instance, previous studies reported that glassy carbon exhibits stopping powers 4–5% higher than diamond for alpha particles below 4 $\textrm{MeV}$^61^. This illustrates the sensitivity of electronic stopping to bonding configurations and local electronic environments. The extracted $I_{\textrm{adj}}$ values from earlier studies are consistent, within uncertainties, with the ICRU-recommended values. The result of Santry *et al.* is closer to the amorphous carbon reference, whereas one dataset from Trzaska *et al.* is more consistent with the graphite value. The 2002 measurement by Trzaska *et al.*^57^ is an exception to this trend.

In the present work, the observed differences in stopping power between graphenic carbon (GC) foils from KETEK and Applied Nanotech are attributed to variations in their chemical composition and microstructure. A systematic comparison of stopping behavior can be achieved by extracting the experimentally adjusted mean excitation energies, $I_{\textrm{adj}}$. As discussed by Ahlen^6^ and the NAS–NRC Subcommittee report^51^, deviations between $I_{\textrm{adj}}$ and the mean excitation energy $I_{\textrm{m}}$ become significant only for large $Z_2$.

The extracted value for the KETEK foil, $I_{\textrm{adj}} = (73\pm 2)\,\textrm{eV}$, lies slightly below the ICRU reference value for graphite $I_{\textrm{m}}=(78 \pm 7)\,\textrm{eV}$^48,49^ and agrees, within uncertainties, with optical spectroscopy measurements for graphite $I_{\textrm{m}}(77 \pm 4)\,\textrm{eV}$^50^. This indicates that the electronic structure of the KETEK material is predominantly graphitic, consistent with a largely $sp^2$-hybridized structure resulting from the CVD-based manufacturing process^14^.

In contrast, the elevated $I_{\textrm{adj}}$ values obtained for the Applied Nanotech foils are associated with their non-stoichiometric composition (89.6 at.% carbon, 8.8 at.% oxygen, and 1.6 at.% nitrogen)^23^. Furthermore, Haubner *et al.*^62^ demonstrated that carboxyl (–COOH) groups persist after chemical reduction, resulting in a stable residual oxygen content. These oxygen-related functional groups modify the local electronic structure and excitation spectrum, thereby increasing the effective mean excitation energy. The fitting procedure yields an adjusted mean excitation energy for the carbon component of $I_{\textrm{adj}}(\textrm{C}) = (83 \pm 3)\,\textrm{eV}$, which aligns closely with the ICRU-recommended value for amorphous carbon $I_{\textrm{m}}=(81 \pm 7)\,\textrm{eV}$^48,49^.

### Non-destructive areal-density determination

Building on the fitted stopping-power model, the alpha-particle energy-loss method provides a practical, non-destructive approach for determining the areal density of GC foils. In this context, non-destructive means that the measurement does not require cutting, dissolving, weighing after removal, or otherwise consuming the foil. Dedicated post-irradiation AFM, TEM, or Raman characterization was not performed to exclude subtle structural modifications; therefore, the method could be described precisely as non-material removal under the low-activity alpha-source conditions used in this work.

For foils with areal densities around 0.2 $\mathrm {mg\,cm^{-2}}$, the reconstructed values are statistically consistent with the reference mass-and-area measurements for the two foils investigated. The use of a three-isotope mixed alpha source provides several independent energy-loss points, enabling an internal consistency check over the investigated energy range.

## Conclusion

This work demonstrates a proof-of-concept alpha-particle energy-loss method for determining the areal density of graphenic carbon foils without material removal. High-resolution alpha spectroscopy in the 5.0–5.8 $\textrm{MeV}$ range enabled energy-loss measurements with sub-percent relative uncertainty. For the two reference foils investigated, the areal densities obtained from the energy-loss method, $0.185 \pm 0.001\,\mathrm {mg\,cm^{-2}}$ for KETEK and $0.193 \pm 0.001\,\mathrm {mg\,cm^{-2}}$ for Applied Nanotech, agree with the corresponding mass-and-area values within uncertainty.

The stopping-power data were consistently described using a Bethe formalism including Barkas and Bloch corrections with an adjusted mean excitation energy. The fitted values, $I_{\textrm{adj}}=(73\pm 2)\,\textrm{eV}$ for KETEK and $I_{\textrm{adj}}=(85 \pm 3)\,\textrm{eV}$ for Applied Nanotech, indicate material-dependent effective stopping behaviour within the adopted model; however, since the model parameters are extracted from the same reference foils, these differences should be regarded as model-dependent rather than independently validated.

The present study demonstrates feasibility and internal consistency, but broader validation will require additional foils with independently measured areal density, composition, and structure using the complementary techniques discussed above. The achievable accuracy of both stopping-power and areal-density measurements is ultimately limited not by the alpha-spectroscopic technique itself, but by the quality of the independent target and composition metrology.

## Data Availability

The datasets generated and/or analyzed during the current study are available from the corresponding author upon reasonable request.
